# Adaptations to relational facilitation for two national care coordination programs during COVID-19

**DOI:** 10.3389/frhs.2022.952272

**Published:** 2022-07-22

**Authors:** Heidi Sjoberg, Rachael R. Kenney, Brianne Morgan, Brigid Connelly, Christine D. Jones, Hebatallah Naim Ali, Catherine Battaglia, Heather M. Gilmartin

**Affiliations:** ^1^Rocky Mountain Regional VA Medical Center, VA Eastern Colorado Healthcare System, Aurora, CO, United States; ^2^Division of Hospital Medicine, Department of Medicine, University of Colorado, Anschutz Medical Campus, Aurora, CO, United States; ^3^Heller School for Social Policy and Management, Brandeis University, Waltham, MA, United States; ^4^Colorado School of Public Health, Department of Health Systems, Management and Policy, University of Colorado, Anschutz Medical Campus, Aurora, CO, United States

**Keywords:** implementation strategies, adaptation, care coordination, veterans, COVID-19 impact, relational coordination

## Abstract

**Background:**

Adaptations to implementation strategies are often necessary to support adoption and scale-up of evidence-based practices. Tracking adaptations to implementation strategies is critical for understanding any impacts on outcomes. However, these adaptations are infrequently collected. In this article we present a case study of how we used a new method during COVID-19 to systematically track and report adaptations to relational facilitation, a novel implementation strategy grounded in relational coordination theory. Relational facilitation aims to assess and improve communication and relationships in teams and is being implemented to support adoption of two Quadruple Aim Quality Enhancement Research Initiative (QA QUERI) initiatives: Care Coordination and Integrated Case Management (CC&ICM) and the Transitions Nurse Program for Home Health Care (TNP-HHC) in the Veterans Health Administration (VA).

**Methods:**

During 2021–2022, relational facilitation training, activities and support were designed as in-person and/or virtual sessions. These included a site group coaching session to create a social network map of care coordination roles and assessment of baseline relationships and communication between roles. Following this we administered the Relational Coordination Survey to assess the relational coordination strength within and between roles. COVID-19 caused challenges implementing relational facilitation, warranting adaptations. We tracked relational facilitation adaptations using a logic model, REDCap tracking tool based on the Framework for Reporting Adaptations and Modifications-Enhanced (FRAME) with expanded Reach, Effectiveness, Adoption, Implementation, Maintenance (RE-AIM) dimensions, and member checking. Adaptations were analyzed descriptively and for themes using matrix content analysis.

**Results:**

COVID-19's impact within the VA caused barriers for implementing relational facilitation, warranting eight unique adaptations to the implementation strategy. Most adaptations pertained to changing the format of relational facilitation activities (*n* = 6; 75%), were based on external factors (*n* = 8; 100%), were planned (*n* = 8; 100%) and initiated by the QA QUERI implementation team (*n* = 8; 100%). Most adaptations impacted adoption (*n* = 6; 75%) and some impacted implementation (*n* = 2; 25%) of the CC&ICM and TNP-HHC interventions.

**Discussion:**

Systematically tracking and discussing adaptations to relational facilitation during the COVID-19 pandemic enhanced engagement and adoption of two VA care coordination interventions. The impact of these rapid, early course adaptations will be followed in subsequent years of CC&ICM and TNP-HHC implementation.

## Introduction

An implementation strategy is an action completed to promote the adoption, implementation, and sustainment of an evidence-based practice (1). Adaptations to implementation strategies are often necessary to support adoption and scale-up of evidence-based practices in real-world settings. Adaptations are defined as modifications to an implementation strategy to enhance the fit, adoption, feasibility, and acceptability of the implementation strategy in unique contexts (2, 3). Systematically identifying, tracking, reporting, and discussing adaptations can be critical for understanding the impact of an implementation strategy on program outcomes. However, adaptations to implementation strategies are infrequently tracked. Not tracking adaptations to implementation strategies can limit a team's ability to identify what went well, what should be changed or repeated, and how to spread an implementation strategy across programs, settings, and populations.

Implementation of evidence-based practices can be challenging (4) in the best of times. The COVID-19 pandemic has created additional challenges within every sector of the healthcare system. This included restructuring of healthcare delivery to rapidly diagnose, isolate, and care for COVID-19 positive patients while continuing care for acute and chronic conditions. To respond to surges in hospitalizations during the pandemic, many clinical staff have been reassigned or asked to provide care in virtual settings. Many research and quality improvement efforts were curtailed unless directly related to COVID-19 (5). As the pandemic has continued, high rates of healthcare staff turnover has decreased staffing levels, requiring remaining staff to take on additional duties to ensure continuation of care delivery (6). Healthcare providers are reporting high levels of emotional exhaustion, fear, stress, anxiety, and depression (6, 7). The COVID-19 pandemic has required implementation teams to be agile and flexible to support adoption and implementation of evidence-based practices while recognizing the burden the COVID-19 pandemic continues to have on healthcare providers. The purpose of this project was to describe a new method to systematically track and report adaptations to the relational facilitation implementation strategy during the COVID-19 pandemic.

Relational facilitation is a novel implementation strategy that aims to assess and improve communication and relationships within and between teams to support program outcomes. The relational aspect is guided by the theory of relational coordination, which is defined as a mutually reinforcing process of communicating and relating for the purpose of task integration (8). Relational coordination includes a theory and set of analytic methods for understanding the relational dynamics of coordinating work within and between individuals and teams. The theory proposes that when coordination is carried out through frequent, high-quality communication supported by relationships of shared goals, shared knowledge and mutual respect, organizations can more readily achieve their desired outcomes. The relational coordination analytic methods assess coordination within a work process narrowly or broadly defined (e.g., transitions of care for high-risk Veterans or the work we do together), display relationships in the form of a social network map, and assess the strength of ties between roles using specific communication and relationship dimensions (i.e., frequent, timely accurate, problem-solving communication) (9). Adoption of relational coordination-guided interventions has been shown to enhance implementation of three national Veterans Health Administration (VA) care coordination programs (10, 11). Additional research in the VA indicates that relational coordination supports the implementation of new practices as well as employee engagement and the quality of care (12–15). The facilitation aspect of relational facilitation is operationalized as individual members of the implementation team support and enable practitioners to adopt and sustain new practices.

The VA Quadruple Aim Quality Enhancement Research Initiative (QA QUERI) is using relational facilitation to support the implementation of two evidence-based care coordination interventions, Care Coordination and Integrated Case Management (CC&ICM) and/or Transitions Nurse Program-Home Health Care (TNP-HHC). For the purposes of this article we will refer to CC&ICM and TNP-HHC collectively as care coordination initiatives. Briefly, the CC&ICM (16, 17) initiative is a practice change nationally mandated in the VA in 2021, as a collaboration between the VA Offices of Care Management and Social Work and the VA Office of Nursing Services. The main goals of CC&ICM are to standardize and integrate care coordination services across all VA facilities and points of care for complex Veterans (18). Complex Veterans enrolled into CC&ICM are assigned a lead coordinator as a clearly identified single point of contact. CC&ICM is a mandated initiative and will be deployed throughout and across all VAs. In 2020 the QA QUERI partnered with National VA to add a research component to support implementation and evaluate CC&ICM at six VA medical centers. The TNP-HHC is primarily a nurse-led care coordination intervention (but can also be social work-led) that was launched in 2020 and is modeled off the core components from the VA rural Transitions Nurse Program (19, 20). The main goal of this program is to improve care for high-risks Veterans transitioning home from a VA medical center with a focus on Veterans who require home health care services. A nurse or social worker transitions coordinator collaborates with inpatient and outpatient medical teams to address the Veterans medical and social needs to enhance the transitions of care. The QA QUERI currently supports implementation of TNP-HHC at three VA medical centers. The QA QUERI implementation team (which we will refer to as the implementation team) supports implementation of these care coordination initiatives by providing intervention education, resources, creation of a learning community, relational facilitation, and program evaluation using an iterative Reach, Effectiveness, Adoption, Implementation, and Maintenance (RE-AIM) framework (21–23). VA medical centers implementing the care coordination initiatives were rolled out in a stepped-wedge fashion with implementation of the care coordination initiatives occurring in sequential order.

## Materials and methods

We used a new multi-methods approach to track adaptations and analyze data that emerged.

### Relational facilitation study design

Members of the implementation team were trained on the theory and practice of relational coordination during a 3-day relational coordination workshop offered by the Institute for Excellence in Health and Social Systems at the University of New Hampshire. The course included 6 months of coaching by content experts to address barriers and facilitators to implementing relational coordination assessments and interventions in the real world. The relational facilitation strategy was developed, and field tested during the workshop.

Relational facilitation is a multi-step implementation strategy that occurs during pre-implementation, implementation, and sustainment phases. For the purposes of the QA QUERI, relational facilitation begins once a VA medical center enrolls in either of the care coordination initiatives and begins pre-implementation activities. The implementation team initially planned a 2-day in-person workshop to provide education on the theory and practice of relational coordination and group coaching with site stakeholders to create a relational map of all roles that support Veterans enrolled in one of the care coordination initiatives. The workshop concludes with attendees qualitatively rating the strength and quality of relationships and communication between roles, discussing the results and potential next steps. Once the site has begun to enroll Veterans in one of the care coordination initiatives, the site leads are asked to identify individuals within each role listed on the relational map, along with email addresses. The QA QUERI team then invites members of one of the care coordination initiatives site teams to participate in the Relational Coordination Survey.

The Relational Coordination Survey measures relationships and communication as a network of ties within and between roles. The survey is designed to ask respondents to report the behaviors of others as opposed to being asked to report their own behaviors (e.g., “Do people in these groups communicate with you in a timely way…”). The goal is to minimize the problem of self-report or social desirability bias, where respondents tend to overestimate their own socially desirable behaviors (9). The network approach increases the accuracy of measurement for respondents are asked to evaluate connections with each role, not a specific individual in a role. This allows for the diagnosis of strong and weak ties and the drilling down to the level of role dyads within a team (9). The Relational Coordination Survey is administered through the RC Analytics on-line survey platform over a 2-week period during the first 2 months of implementation. Participants are invited to complete the survey once during the implementation phase and once during the sustainment phase. RC Analytics analyzes the survey data and compiles the results in a standardized report.

The implementation team shares the Relational Coordination Survey results with sites during virtual learning sessions and also email the results to sites to identify bright spots and select relational interventions to address gaps in relationships and communication. The results are reviewed in follow-up sessions to develop goals that address gaps identified in the initial survey results session. Active relational facilitation ends once the care coordination initiatives site teams have selected and implemented relational facilitation interventions. Progress monitoring occurs as part of the ongoing work between the implementation team and sites. Relational facilitation is revisited as needed to address interventions that are not working or new challenges that arise.

The goal for relational facilitation is to support teams to become open and adaptable to change and integrate relational coordination as part of their standard practice. This occurs through the internalization of relational coordination attitudes exemplified by frequent, high-quality communication supported by relationships of shared goals, shared knowledge, and mutual respect. Behaviors that indicate successful application of relational facilitation include boundary spanning activities by the care coordination initiatives coordinators at each site, such as proactive problem solving, effective conflict resolution and standardized communication methods. The targeted outcomes for relational facilitation include improved care coordination through adoption and sustainment of one of the care coordination initiatives at sites, engagement and sustained use of relational interventions, and improved scores on the Relational Coordination Survey administered during the sustainment phase at implementation sites.

**Figure 1:**
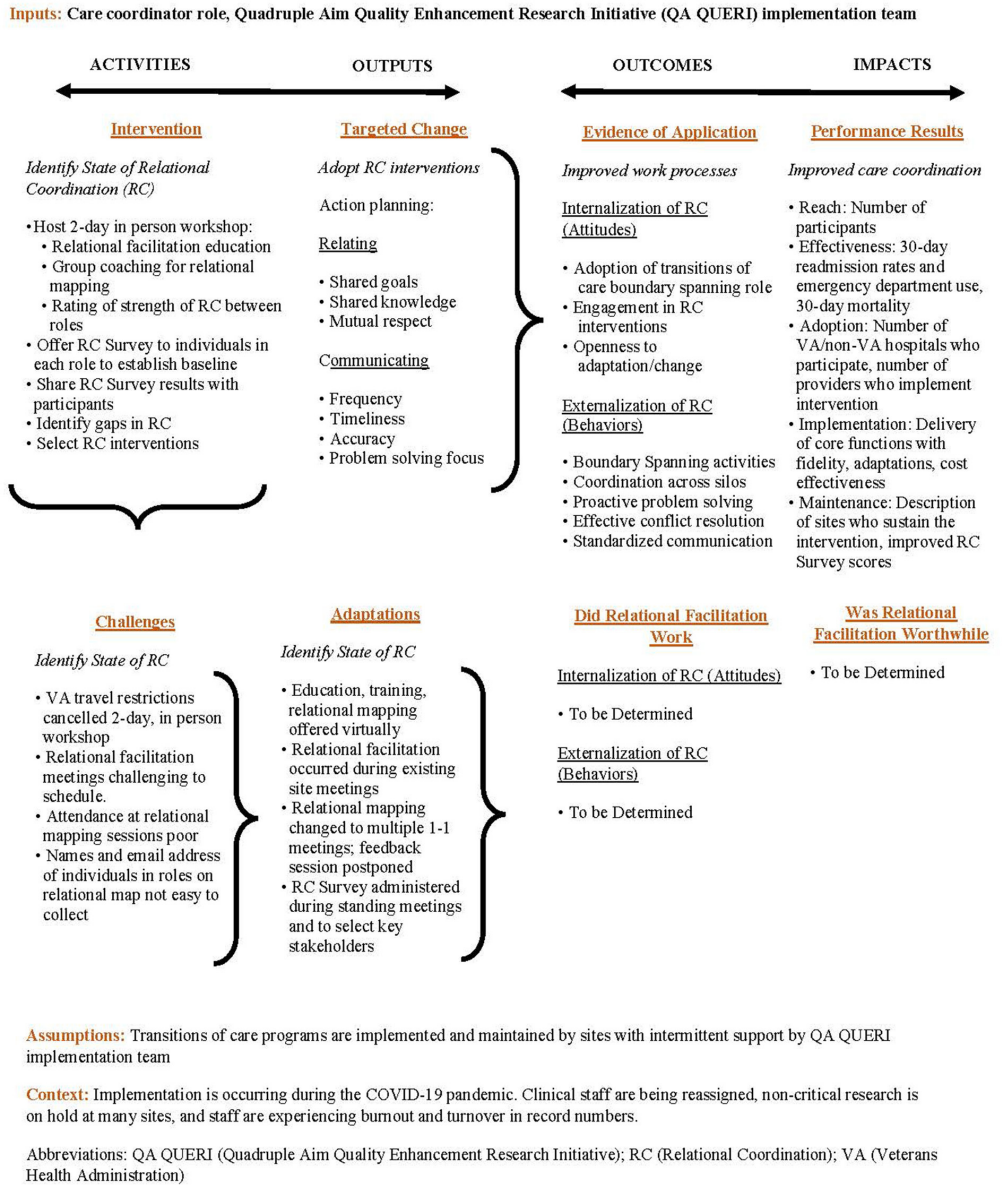
Relational facilitation logic model.

We developed a logic model outlining the above steps for the ideal process of administering relational facilitation and identifying expected outcomes. Logic models provide visual representation of the relationships between an intervention and the intended effects and are created during the pre-implementation phase of a project. Using logic models increases the probability that interventions will be successful as they involve multiple stakeholders responsible for designing the pre-implementation, implementation, and sustainment phases of an intervention (24). Logic models clearly outline the purpose of the intervention, strategies, actions that are expected to lead to desired outcomes, and anticipated outcomes. We described the ideal steps to administer relational facilitation in the first row of the logic model (Figure 1). However, as relational facilitation was being implemented we ran into challenges and added a second row in the logic model to describe the challenges and what relational facilitation components were adapted as a result. This process is outlined in detail below.

### QA QUERI setting and participants

The implementation team launched relational facilitation activities with the care coordination initiatives sites starting in July 2021. The QA QUERI is now collaborating with nine VA medical centers across the United States to implement one of these initiatives. These nine VA medical centers have completed QA QUERI pre-implementation work, including relational facilitation education. Five of the sites have completed relational mapping activities and three have completed Relational Coordination surveys. Implementation of relational facilitation was led by the multidisciplinary implementation team that includes social workers, nurses, a physician, implementation scientists, experts in qualitative and quantitative research, an implementation adaptations specialist, and relational coordination experts. Implementation of the interventions was led by the site teams and supported by the implementation team.

### CC&ICM setting and participants

The site teams for CC&ICM include an executive sponsor/staff, nurse and social worker co-champions, data analysts, group practice managers, information technology specialists, chief nurses and social workers, executive officers, and nurse and social worker consultants from National VA offices.

### TNP-HHC setting and participants

The site teams for TNP-HHC consist of nurses, nurse managers, social workers, executive officers, associate directors, deputy associate directors, and chief nurses and chief social workers.

### Adaptations tracking and analysis

Relational facilitation adaptation tracking and analysis occurred through multiple methods to corroborate findings and identify any weaknesses, allowing for data triangulation. These methods include updating our previously described logic model, an adaptations database, and member checking meetings.

As previously mentioned, the relational facilitation logic model was developed during the pre-implementation phase to outline the planned intervention, targeted change, evidence of application and performance results (activities, outputs, outcomes, and impacts). During the implementation phase, when the implementation or clinical teams experienced challenges and/or adaptations were made, they were discussed during monthly implementation meetings and documented in the logic model (Figure 1).

Adaptations were tracked in a REDCap hosted tracking tool, based on the Framework for Reporting Adaptations and Modifications-Enhanced (FRAME) expanded with RE-AIM dimensions (2, 3). This tool is currently being piloted with the care coordination initiatives. Adaptation data from all sites were collected and entered by the QA QUERI implementation clinical leads (implementation clinical leads) in real-time. After the data were entered, the implementation clinical leads consulted the implementation team and our adaptations specialist to discuss adaptations and resolve discrepancies until consensus was reached. The data were downloaded and analyzed descriptively based on the FRAME and RE-AIM dimensions.

Member checking was conducted during implementation team monthly meetings to review and verify adaptations, resolve discrepancies, and discuss the potential impact of adaptations on intervention processes and outcomes. Meetings included the implementation team, implementation clinical leads, our adaptations specialist, and our implementation scientist.

Data triangulation provided a richer understanding of adaptations by comparing data sources. We triangulated data by comparing documented adaptations from our multi-methods (logic model, REDCap tracking tool and member checking) to understand similarities and differences and to expand on identified adaptations. Adaptation themes were identified based on the FRAME and RE-AIM dimensions using matrix content analysis. Adaptations were inductively coded using this method where data was abstracted from our documented adaptations and listed under pre-defined categories identified from the FRAME and RE-AIM. Adaptations were reported quarterly to QA QUERI leadership to discuss actual impacts on the care coordination initiatives processes and outcomes. The QA QUERI activities are undertaken in support of a VA operational project and do not constitute research as defined by the VA Handbook 1058.05. Therefore, institutional review board approval was not required.

## Results

The COVID-19 pandemic overlapped with the QA QUERI pre-implementation and implementation phases. As a result, many care coordinators and clinical teams were redeployed to support the COVID-19 response and site leaders from VA medical centers that had committed to participating with CC&ICM and TNP-HHC were unable to dedicate staff and protected time to these interventions. During this time many non-COVID-19 related VA quality improvement programs, such as CC&ICM and TNP-HHC were placed on hold. In 2021 sites began pre-implementation work. However, travel was restricted and sites reported challenges identifying current staff to take on the role of CC&ICM lead coordinator or TNP-HHC transitions coordinator. Further, these staff were not provided dedicated time for pre-implementation activities due to short staffing and turnover across VA medical centers. As a result, the implementation team made eight unique adaptations to the relational facilitation implementation strategy.

The timing of adaptations by sites was dependent on the stepped-wedge approach of the care coordination initiatives adoption. The first sites to implement the care coordination initiatives reported one relational facilitation adaptation (13%) during pre-implementation and seven (88%) during early implementation. The second TNP-HHC site reported seven adaptations during pre-implementation (88%), and one during implementation (13%). The second through sixth sites to implement CC&ICM and third site to implement TNP-HHC reported eight adaptations, all occurring during the pre-implementation phase (Table 1).

**Table 1:** Relational facilitation adaptations by site and implementation phase.

**Site**	**Pre-implementation**	**Implementation**
	***N*** **(%)**	***N*** **(%)**
**TNP-HHC**		
Site 1	1 (13)	7 (88)
Site 2	7 (88)	1 (13)
Site 3	8 (100)	0 (0)
**CC&ICM**		
Site 1	1 (13)	7 (88)
Site 2	8 (100)	0 (0)
Site 3	8 (100)	0 (0)
Site 4	8 (100)	0 (0)
Site 5	8 (100)	0 (0)
Site 6	8 (100)	0 (0)

**Figure 2:**
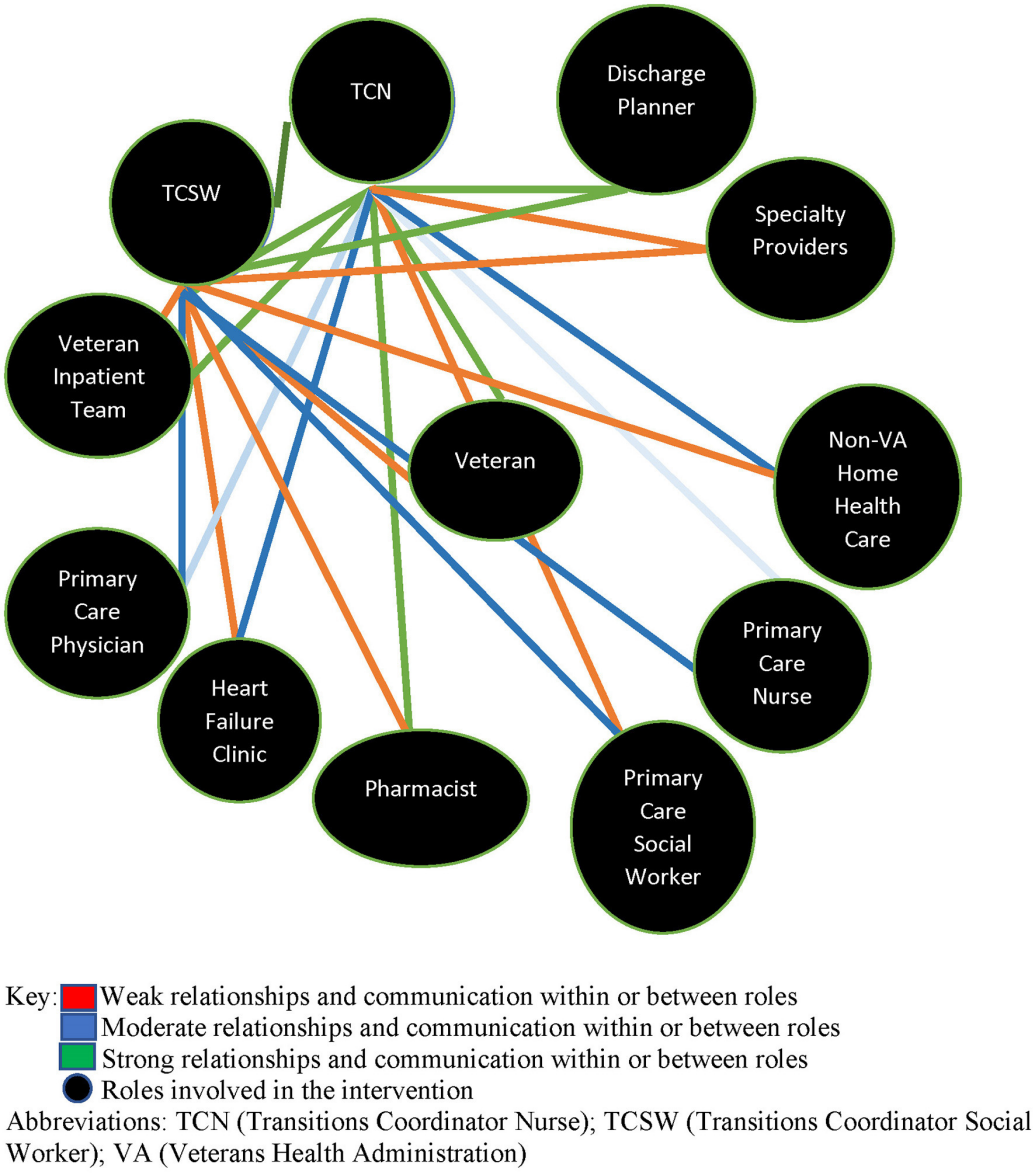
Relational map for transitions nurse program-home health care site one.

Initial adaptations to relational facilitation included canceling the two-day, in person workshops and weaving the education, training, and relational mapping work into existing meetings between the implementation clinical leads and staff implementing the care coordination initiatives. Relational coordination and relational facilitation education was provided by the implementation clinical leads virtually using videos or with presentations to site teams and recordings of the presentations were made available on a VA website for independent learning (25). The relational mapping exercise was adapted from a large group exercise to a 1-on-1 or small group discussion between the implementation clinical leads based out of the Denver VA and the care coordination initiatives staff based out of their respective site locations. The site-specific relational maps were pre-built by the implementation clinical leads with roles identified during pre-implementation process mapping and site interviews to visually represent the ideal care coordination initiatives site teams. Due to intermittent attendance by site staff at standing meetings the relational maps were reviewed for role alignment and the strength and quality of relationships and communication between roles during multiple meetings with individual site staff (Figure 2).

Administration of the Relational Coordination Survey also required multiple adaptations. First, implementation clinical leads, the care coordination initiatives staff and site leadership found the identification of multiple individuals within each role on the care coordination initiatives relational maps challenging. In some cases, site leads shared they were aware of individuals in a role but were reluctant to provide contact information for they worried the survey would burden staff who were already overwhelmed with regular duties and responding to COVID-19. To address this challenge, the implementation clinical leads reviewed corollary data from CC&ICM Site One and TNP-HHC Sites One and Two to identify providers who functioned in roles identified on the relational map. Provider email addresses were then identified through the National VA Address Book. However, this required 3–4 h of work per site and was deemed too time intensive for future sites (Table 2).

**Table 2:** Relational facilitation adaptations: descriptive.

**Planned relational facilitation delivery**	**Adapted relational facilitation**
Two day in-person workshop with relational coordination and relational facilitation education and group relational mapping exercise.	Weaved education, training, and relational mapping into existing site meetings.
	Education provided virtually using videos or brief presentations.
	Posted educational content on VA website for independent learning.
	Relational mapping occurred during existing site meetings as 1-on-1 exercise using pre-build relational map.
	Review of strengths and quality of relationships and communication between roles occurred over multiple meetings with individual staff.
	Relational map results review postponed.
**Relational Coordination Survey**	
Request names and email addresses for individuals in roles from site leads	Conduct additional review to identify individuals engaged in programs. Collect emails from National VA Address Book.
Administer survey *via* email	Relational Coordination Survey completed during standing meetings through a link provided in the chat box
Key stakeholders who do not attend meetings emailed Relational Coordination Survey link by the implementation team	Stakeholders sent link by program site leads.

To simplify the survey process, the care coordination initiatives site staff were asked to complete the Relational Coordination Survey during standing meetings through a link provided in the chat box. Key stakeholders who did not attend meetings and were deemed important voices to capture were emailed a link to the survey by the implementation team at TNP-HHC Site One. However, no stakeholders completed the survey. As a result, at TNP-HHC Site Two, the site leads emailed the request to complete the Relational Coordination Survey, which increased response rates. The adaptations were captured in the Relational Facilitation Logic Model (Figure 1) after member checking with the implementation team and site leads. The Relational Coordination Survey data reporting and feedback plans required no adaptations.

### Adaptation themes

We used matrix content analytic methods to identify and analyze themes that emerged across adaptations mapped to the FRAME constructs and RE-AIM dimensions (Table 3). The denominator for our analysis is the total number of adaptations under each FRAME construct. Analysis of the FRAME constructs conducted by the implementation team indicated the format of relational facilitation was adapted six times (75%), while personnel involved, the target population and the intervention presentation were each adapted once. The type of change was primarily substitution for a component of relational facilitation (*n* = 6; 75%), though extending a component and changing the intervention were both adapted once. All adaptations were initiated by the implementation team. The basis for the changes were largely for pragmatic or practical considerations (*n* = 6; 75%), while two (25%) were due to feedback or suggestions and one was due to changes in contracting with RC Analytics. Member checking indicated the pragmatic reasons for adaptations were driven by the challenges imposed by the COVID-19 pandemic and the competing demands placed on the care coordination initiatives site staff during implementation.

**Table 3:** FRAME and expanded RE-AIM adaptations to relational facilitation.

**FRAME adaptation constructs**	**Total (** ***N*** **)**
**Elements that were changed**	
Format	6
Personnel involved	1
Target population	1
Intervention presentation	1
**Type of change**	
Extending a component	1
Substituting for a component	6
The intervention	1
**Who initiated this modification**	
Other: implementation team	8
**Basis for change**	
Pragmatic/practical considerations	6
Feedback or suggestions	2
Other: RC Analytics offered to do the Relational Coordination Survey	1
**RE-AIM dimension**	
Adoption	6
Implementation	2
**Was the adaptation a result of external or internal issues**	
External issues	8

Analysis by the implementation team of the RE-AIM dimensions indicated that 6 (75%) of the 8 adaptations were made to enhance site adoption of relational facilitation activities. Two (25%) adaptations were made to impact the implementation of the care coordination initiatives interventions. All eight adaptations were a result of external issues, specifically the challenges staff were experiencing during the COVID-19 pandemic. Key themes that emerged during member checking revealed that the most impactful external issue was the COVID-19 related travel restriction, which required all relational facilitation activities to be moved from an in-person 2 day workshop to a virtual environment. Additional adaptations related to minimizing the time burdens of clinical staff, so they could fully participate in relational facilitation at their own pace without additional meetings during or after their regular work shifts.

## Discussion

### Summary

Systematically tracking and discussing adaptations to relational facilitation using a multi-method, theoretically guided approach enhanced adoption and implementation of the care coordination initiatives during the COVID-19 pandemic.

Guidance and step-by-step frameworks on how to track and report adaptations have been published (3, 26) and applied to settings including community implementation of mental health best practices (27), chronic disease prevention best practices (28), and autism mental health practices for Latinx families (29).

The contribution to the literature from this work is the multi-method approach that facilitated triangulation of adaptation data to enhance the validity and reliability of findings.

The logic model method documented the planned intervention, targeted changes, evidence of application and performance results along with the rationale behind specific challenges and adaptations. The logic model provided unique data that enhanced our understanding of implementing relational facilitation and communication progress with QA QUERI leadership. This method required multiple meetings during the pre-implementation phase to finalize the initial logic model, but was an easy-to-use method for discussing, tracking and reporting adaptations during implementation. The adaptations tracking tool, mapped to the FRAME and expanded RE-AIM dimensions (3, 23) facilitated real-time documentation, reporting, and analyses of adaptations. This method required significant investment in time and expertise to develop but will become an open access tool for teams new to implementation science. Member checking provided rich contextual data that were not collected through the logic model or adaptation tracking tool and ensured all team members were engaged in program implementation and adaptation. Member checking was integrated into standing meetings and was acknowledged as an important communication tool to bring all team members to consensus. Member checking provided a forum for our team to clarify what constitutes an adaptation and resolve discrepancies about documented adaptations. Independently, these methods add value to adaptation tracking. However, combined they enhance the validity and reliability of our findings.

The methods described in previous studies were primarily retrospective, qualitative approaches. The adaptation tracking method developed for the care coordination initiatives and relational facilitation was real-time tracking by those doing the work. This approach maximized the fit between the relational facilitation implementation strategy and the care coordination initiatives. The adaptations tracking process, along with evaluation through member checking and documentation in a logic model ensured the implementation team could spread and scale-up relational facilitation, with fidelity, across multiple sites. The impact of these rapid, early course adaptations will be followed in subsequent years of the care coordination initiatives implementation.

### Strengths and limitations

Strengths of this study included the multi-methods approach (i.e., logic model, REDCap tracking tool, and member checking) to tracking, evaluating, and reporting adaptations. This supported timely and rich data collection and enhanced relational facilitation fidelity through triangulation of data between clinical leads and relational coordination experts during member checking. Limitations included time constraints among QA QUERI team members as everyone works on multiple projects and often have more than one role on each project (i.e., an implementation clinical lead provides both clinical guidance to sites and also functions as the relational facilitation lead for sites). Utilizing multi-methods was a limitation as it was more time consuming. However, multi-methods enhanced the rigor of our approach and provided richer data, increasing the understanding of our adaptations. Further, the implementation team continually discusses what constitutes an adaptation to an evidence-based implementation strategy vs. an adaptation to an intervention, leading to potential reporting bias.

## Conclusion

Contextually sensitive adaptations to implementation strategies are essential to successfully adopt evidence-based interventions. This study contributes to the implementation science adaptation literature through the rigorous reporting of a real-time tracking approach which allowed clinical leads to easily report adaptations followed by member checking, which enabled discussion regarding when, to what extent and how adaptations were working. This work was especially critical during the perpetually changing context of the COVID-19 pandemic.

## Data availability statement

The raw data supporting the conclusions of this article will be made available by the authors, without undue reservation.

## Author contributions

HS, RK, BM, BC, CJ, HA, CB, and HG jointly designed the study. HS and HG conducted the analyses. All authors drafted the paper. All authors contributed to the article and approved the submitted version.

## Funding

HG was supported by Career Development Award Number 1IK2HX002567-01A1 from the United States Department of Veterans Affairs Health Services Research & Development Service of the VA Office of Research and Development. This work was funded by the VA Quality Enhancement Research Initiative through grant QUE 20-013.

## Conflict of interest

The authors declare that the research was conducted in the absence of any commercial or financial relationships that could be construed as a potential conflict of interest.

## Publisher's note

All claims expressed in this article are solely those of the authors and do not necessarily represent those of their affiliated organizations, or those of the publisher, the editors and the reviewers. Any product that may be evaluated in this article, or claim that may be made by its manufacturer, is not guaranteed or endorsed by the publisher.

## Author disclaimer

The views expressed in this article are those of the author(s) and do not necessarily represent the views of the Department of Veterans Affairs.

## Abbreviations

QA QUERI: Quadruple Aim Quality Enhancement Research Initiative
CC&ICM: Care Coordination and Integrated Case Management
TNP-HHC: Transitions Nurse Program-Home Health Care
VA: Veterans Health Administration
FRAME: Framework for Reporting Adaptations and Modifications Enhanced
RE-AIM, Reach, Effectiveness, Adoption, Implementation: and Maintenance.
